# Stable continual learning through structured multiscale plasticity manifolds

**DOI:** 10.1016/j.conb.2021.07.009

**Published:** 2021-08-17

**Authors:** Poonam Mishra, Rishikesh Narayanan

**Affiliations:** Cellular Neurophysiology Laboratory, Molecular Biophysics Unit, Indian Institute of Science, Bangalore 560012, India

## Abstract

Biological plasticity is ubiquitous. How does the brain navigate this complex plasticity space, where any component can seemingly change, in adapting to an ever-changing environment? We build a systematic case that stable continuous learning is achieved by structured rules that enforce multiple, but not all, components to change together in specific directions. This rule-based low-dimensional plasticity manifold of permitted plasticity combinations emerges from cell type–specific molecular signaling and triggers cascading impacts that span multiple scales. These multiscale plasticity manifolds form the basis for behavioral learning and are dynamic entities that are altered by neuromodulation, metaplasticity, and pathology. We explore the strong links between heterogeneities, degeneracy, and plasticity manifolds and emphasize the need to incorporate plasticity manifolds into learning-theoretical frameworks and experimental designs.

## Introduction

**Figure 1:**
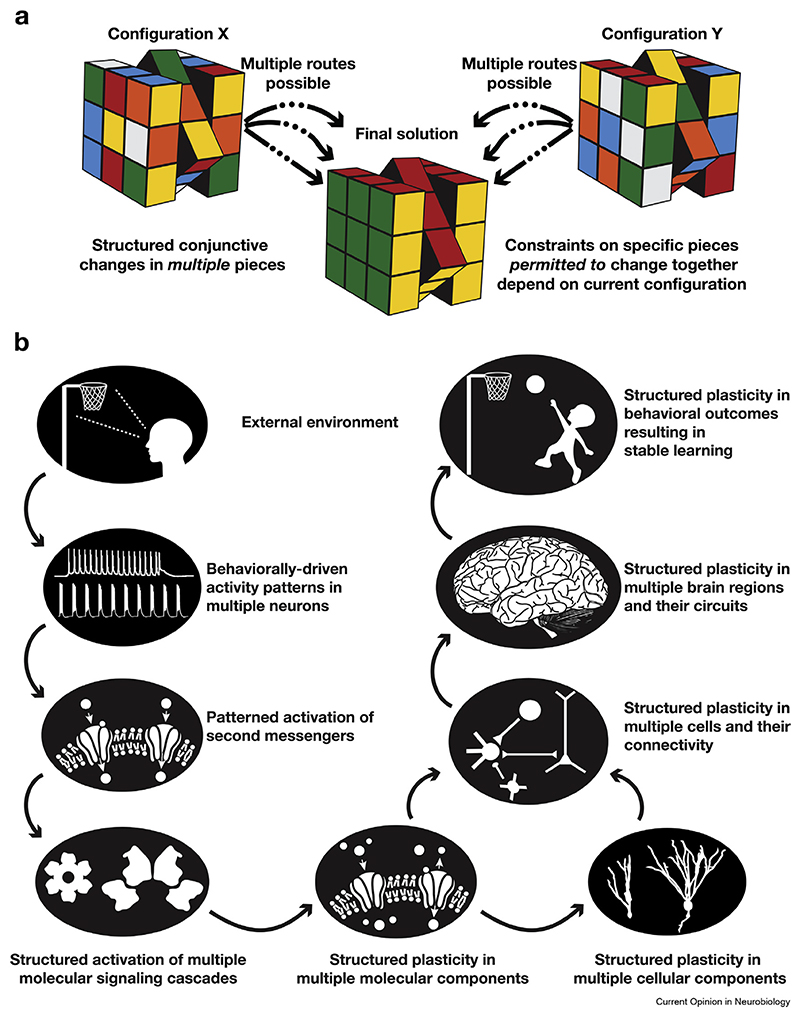
Rubik’s cube puzzle as an analogy for illustrating the structured configuration-dependent conjunctive changes in multiple components that constitute plasticity manifolds. **(a)** There is a single valid solution to Rubik’s cube puzzle, where each face displays a unique color. When the cube’s pieces are analyzed individually, it appears that changes are ubiquitous. However, when movements of multiple pieces are tracked simultaneously, it becomes evident that multiple, but not all, components change together in each step. Importantly, there are strong structured rules, enforced by the current configuration (X vs. Y) of the cube, that permit only certain combinations of pieces to change together. There are several sequences of changes that could yield the final solution, all of which should respect the specific variant of the cube puzzle (e.g. differences in number of sides) and not get entangled in scenarios where solving one side would disrupt the other(s). **(b)** Schematic representation of multiscale plasticity manifolds. Analogous to Rubik’s cube puzzle, independently viewed, plasticity might look ubiquitous, but there are structured rules governing plasticity.

Plasticity is ubiquitous in the brain, with lines of evidence suggesting that changes can occur in any component that governs brain physiology [1]. However, akin to Rubik’s cube puzzle (Figure 1a), the ability of each component to change does not translate to independent random changes in individual components. Instead, there are strong structured rules that permit only certain components to change together. We consider stable adaptation to continually changing environmental stimuli as the ultimate goal of learning-driven plasticity, where learning and homeostasis are achieved without cross-interferences from each other (stable learning) and without catastrophic forgetting of prior learning (continual learning) [2,3]. In this review, we build a systematic case that this ultimate goal of brain plasticity is achieved through structured rules that govern the ability of multiple, but not all, components to change concomitantly. These rules are enforced by the current state of the components and the nature of stimuli and permit only certain combinations of these components to undergo plasticity. We refer to the low-dimensional manifold of permitted plasticity combinations, within the high-dimensional space involving all possible changes spanning all components, as a plasticity manifold. The framework of plasticity manifolds is inspired by the well-established neural manifold framework, which is restricted to represent the rules that govern the population dynamics of correlated firing in interconnected neurons [4–7]. Plasticity manifolds, on the other hand, represent the strong rules that govern conjunctive long-term plasticity in multiscale components and measurements, geared toward adaptation to an altered environment (Figure 1b).

## Emergence of multiscale plasticity manifolds

**Figure 2:**
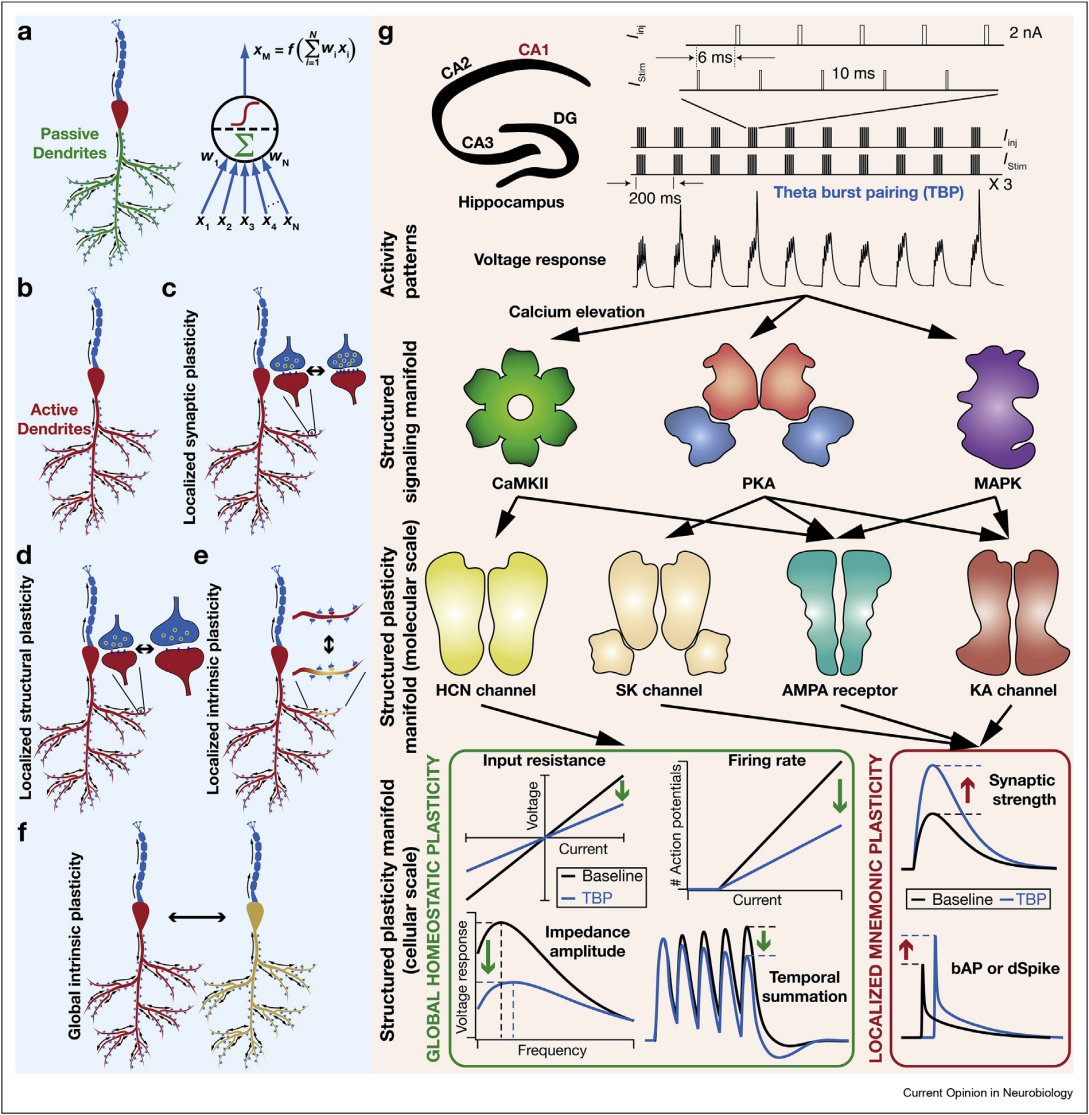
(**A**-**F**) Neurons endowed with active dendrites are powerful computational devices, and plasticity is ubiquitous. **(a)** Several learning-theoretical frameworks use distributed processing by well-connected integrate-and-fire ‘neurons’, which learn through modifications in their ‘synapses’. **(b)** The integrate-and-fire approximation of neurons is contingent on the assumption that neuronal dendrites are passive and house only synaptic receptors. Active dendrites extend single-neuron function beyond passive integration, allowing dendritic spike initiation, bidirectional flow of intraneuronal information, location-specific filtering, and coincidence detection. **(c**–**f)** Learning-induced plasticity is not confined to synaptic weights, but is ubiquitous with different loci of plasticity, **c**: changes in numbers of receptors and vesicles; **d**: changes in size of the spine and the terminal; **e-f**: changes in intrinsic components (e.g. ion channels) confined to a single dendritic branch (**e**) or manifesting globally (**f**). Note that there are global forms of synaptic (e.g. synaptic scaling) and structural plasticity as well. **(g)** The TBP protocol as an example for the emergence of molecular- and cellular-scale plasticity manifolds. The TBP protocol was initially developed to induce robust synaptic plasticity in hippocampal synapses. TBP elicits cytosolic calcium influx, which differentially activates CaMKII, PKA, and MAPK (structured signaling manifold, a specific subset of the several signaling cascades) depending on the strength of the TBP protocol [19–25,49]. These enzymes, in turn, induce changes in AMPARs, HCN, SK, and KA channels (structured molecular-scale plasticity manifold). The consequent cellular-scale plasticity manifold involves concomitant localized increases in synaptic strength, back-propagating action potentials, and dendritic spikes, accompanied by global reduction in sub- and supra-threshold excitability, elicited in response to the same protocol. Note that the same signaling molecule (e.g. PKA) conjunctively induces plasticity in multiple molecular-scale components (AMPARs, KA, and SK channels), which in turn change multiple cellular-scale measurements (synaptic strength and local excitability). These observations show that only a very specific subset of components is permitted to change together in specific directions, and such changes are restricted to specific locations.

Theoretical and computational frameworks that consider neurons as simplified computational units with synaptic plasticity as the substrate for learning (Figure 2a) have a long and cherished history [8,9]. However, most of these theories predate the discovery of active dendrites (Figure 2b), which transform single neurons into powerful computational machines [10,11], and active glial signaling [12–14]. Furthermore, as learning-induced biological plasticity is ubiquitous [1,15–18] (Figure 2c-f), the strong constraints imposed by plasticity manifolds are essential in avoiding disruptive changes (Figure 1).

**Figure 3:**
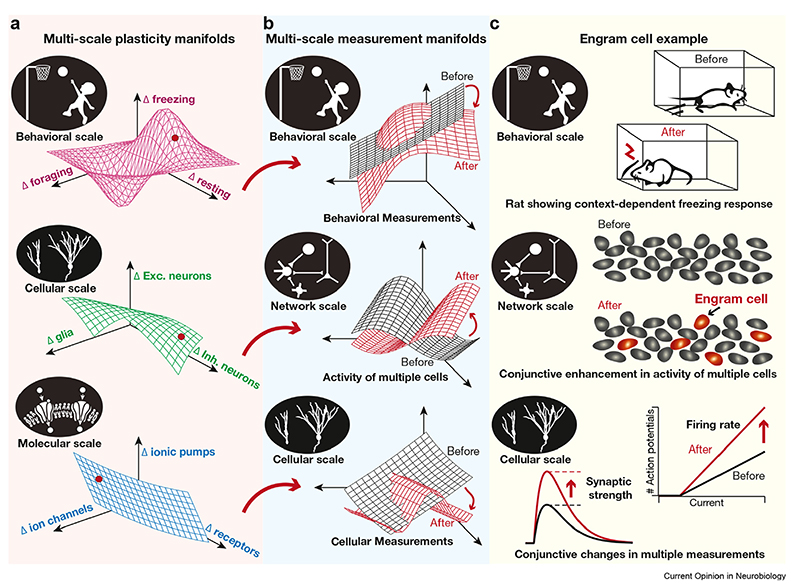
Illustration of multiscale plasticity manifolds from the perspective of engram cell formation. The panels represent the multiscale plasticity manifolds (**a**), measurement manifolds (**b**), and an illustrative example on engram cell formation involving conjunctive multiscale changes in multiple components (**c**). **(a)** In learning tasks, there are molecular- (bottom) and cellular-scale (middle) plasticity manifolds that collectively result in changes in specific behavioral measurements (top). The large red dot in each plasticity manifold represents a specific instance of such conjunctive changes, leading to the adaptation in the measurement manifolds represented in panel B. It is important to note that several such instances (several dots at different locations) could yield the same adaptation in the measurement manifolds (i.e. the same ‘before’ to ‘after’ transitions shown in **b**). **(b)** The components at the cellular (bottom), network (middle), and behavioral (top) manifolds that define the system are subjected to long-term changes (indicated as ‘before’ to ‘after’ in each sub-panel) as a consequence of the implementation of respective plasticity manifolds (red arrows from respective panels in A). The panels show adaptation-induced changes in a manifold of behavioral measurements (top), cellular activity of both neurons and glia (middle), and single-cell measurements (bottom), with each manifold (both before and after configurations) spanning different stimulus conditions. The overlap between the ‘before’ and ‘after’ manifolds represents changes in certain measurements but not others. The cellular-scale manifold here (middle) is a superset of the neural manifolds, in that this accounts for ‘activity’ in neurons and glia. **(c)** Conjunctive changes in several ion channels and receptors at the molecular scale introduce context-specific cellular-scale changes, increased synaptic strength, and enhanced firing rate, in a subset of cells (bottom). These changes alter the response properties of a specific subset of cells, called engram cells, thereby constituting a network-scale plasticity manifold involving changes in activity patterns of multiple cells (middle). The specific subset of cells (not all) that are transformed to engram cells for a given context/learning task is constrained by several factors, including the baseline intrinsic excitability [18,26,28–33]. Finally, engram cells drive behavioral-scale changes, altering the freezing response of the animal in a context-dependent manner (top).

A well-formed example of structured multiscale plasticity manifolds is the theta-burst pairing (TBP) protocol in hippocampal pyramidal neurons (Figure 2g). The cytosolic calcium influx induced by TBP activates a specific subset of downstream signaling cascades, each inducing conjunctive plasticity in specific ion channels and receptors. These molecular-scale changes concomitantly induce localized increases in synaptic strength, back-propagating action potentials and dendritic spikes, accompanied by a global reduction in sub- and suprathreshold excitability [19–25], together yielding a cellular-scale plasticity manifold (Figure 2g). In the hippocampus, multiscale plasticity manifolds are involved in the emergence of a subpopulation of engram cells, through specific combinations of synaptic and intrinsic plasticity [18,26,27], driving context-dependent behavioral changes (Figure 3c). Here, baseline neural excitability plays a critical role in permitting specific subsets of cells to become engram cells and be part of the network-scale plasticity manifold [18,26,28–33]. In the suprachiasmatic nucleus, a network of specific genes mediates the day–night rhythms in excitability properties of neurons. These rhythms recruit plasticity manifolds involving a specific subset of ion channels [34,35] that dramatically alter cellular, network, and behavioral physiology [34–36]. Importantly, the cellular-scale plasticity manifolds in circadian rhythm generation and memory formation also involve glia [12,14,18,26,27,36,37]. Similar examples of multiscale plasticity manifolds are found across different brain regions [1,16,38–41].

As biological plasticity invariably recruits the activation of biochemical signaling cascades, the molecular scale forms the lynchpin in the emergence of plasticity manifolds. The strength and dynamics of signaling species, including cytosolic calcium, activate a specific subset of downstream signaling cascades [42–44]. Once activated, dynamical interactions between these signaling cascades, along with their specific target molecules, regulate the molecular-scale plasticity manifold [42,43,45–48]. The impact of these signaling cascades on each molecular substrate results in gain or loss of function of that substrate, together yielding specific changes in cellular, network, and behavioral-scale function (Figure 3). The continual dependence of the strength and direction of different forms of plasticity on cytosolic calcium and on the graded activation of different signaling molecules constitutes the prime motivation for the framework of a manifold considered here [19,21,49–53].

The rules associated with plasticity manifolds should not be generalized across different cell types or different contexts. For instance, activation of group 1 metabotropic glutamate receptors results in depression of synaptic strength combined with an enhancement of intrinsic excitability [21] in CA1 pyramidal neurons, but induces concomitant enhancement of synaptic strength and intrinsic excitability in amygdalar neurons [54]. Theta-burst firing reduces sub- and supra-threshold excitability through changes in HCN channels in CA1 pyramidal neurons [19,22], but enhances suprathreshold excitability and reduces sub-threshold excitability through conjunctive changes in HCN, inward-rectifier potassium, and persistent sodium channels in dentate gyrus granule cells [40]. Phosphorylation of AMPARs increases AMPAR-mediated current in hippocampal pyramidal neurons, but reduces the current in cerebellar Purkinje cells [55] as a consequence of the differential expression of AMPAR subunits. Thus, it is important that cell type specificity of molecular and cellular plasticity manifolds is explicitly accounted for [19,22,34–36,38,40,54–58].

## Degeneracy, heterogeneities, and plasticity manifolds

**Figure 4:**
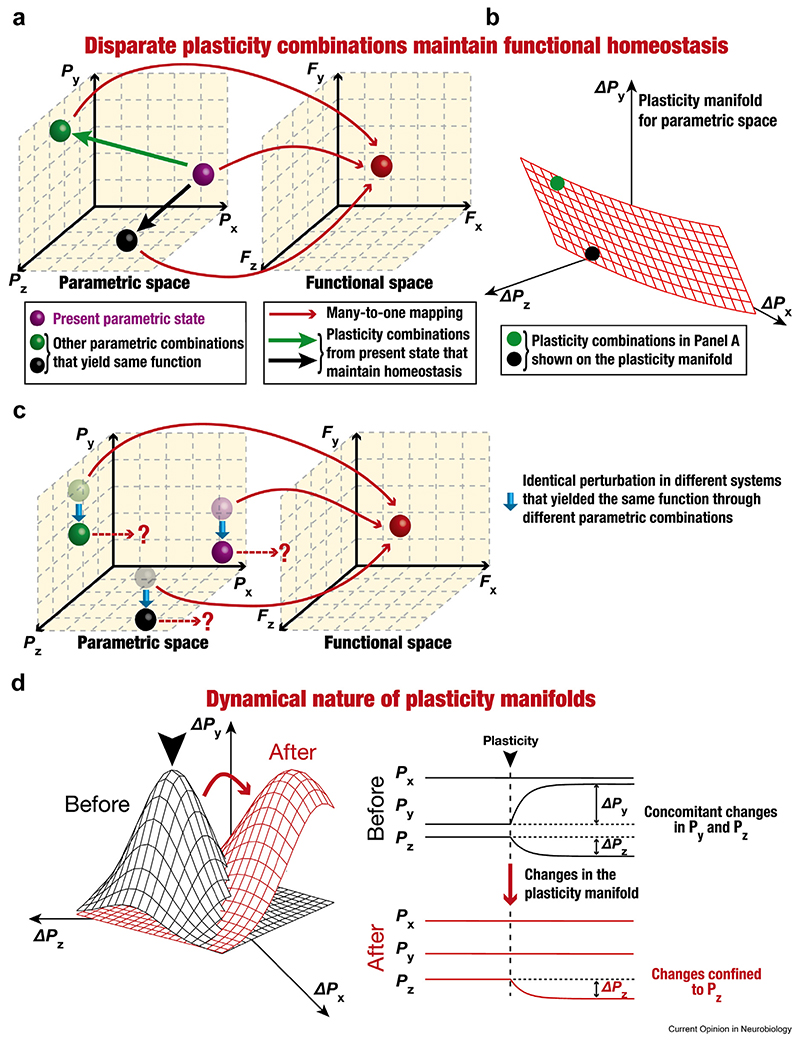
Illustration of the relationship between degeneracy and plasticity manifolds and the dynamical nature of plasticity manifolds. **(a**–**b)** The expression of degeneracy implies that several parametric combinations (green, black, and purple spheres in the parametric space spanning *P_x_, P_y_*, and *P_z_*) yield the same function (red sphere in the functional space spanning *F_x_, F_y_*, and *F_z_*), thereby forming a many-to-one mapping between the parametric and functional spaces (red arrows across the two spaces). Consider the purple sphere to constitute the present parametric state of the system. In the face of perturbations, functional homeostasis could be achieved in this system through transitions to the black or the green spheres. Such transitions require structured changes in multiple parameters, thus recruiting specific plasticity combinations on the plasticity manifold (either the green or the black arrows in panel **a**; also represented as green and black circles in panel **b**). Furthermore, although all three parametric combinations yield the same function, the specific plasticity combinations required to achieve functional homeostasis are dependent on the present state of the system (say, green vs. purple spheres in panel **a**). **(c)** The expression of degeneracy implies that different systems facing the same perturbation would react differently. Consider three distinct systems (transparent spheres in green, purple, and black in the parametric space) yielding the same function (red sphere in the functional space). Now consider an identical artificial perturbation (downward cyan arrow along the *P_y_* axis of the parametric space) to affect all these three systems (respective solid spheres). These off-manifold perturbations yield distinct functional outcomes because they are in different locations in the parametric space. **(d)** Plasticity manifolds are dynamic entities and can change in response to neuromodulation, metaplasticity, or pathological conditions. Left, the plasticity manifolds before (black) and after (red) such changes are shown. Right, concomitant plasticity along the *P_y_* and *P_z_* axes of the parametric space was permitted before changes to the plasticity manifold, whereas permitted changes were confined to the *P_z_* axis after the plasticity manifold changed. The black arrowhead (in both left and right panels) points to a specific location on the manifold. As an example of alteration in cellular-scale plasticity manifolds, in wild-type mice, TBP results in changes to synaptic strength and to neural excitability through changes in HCN channels (Figure 2g). However, in *fmr1^−/y^* mice, TBP results in enhanced synaptic strength, but not in changes to neural excitability [84]. Thus, in wild-type mice, the manifold involved changes in both synaptic and intrinsic properties, but in the mutant mice, there is change in the plasticity manifold. With reference to networkscale plasticity manifolds, considering the example of engram cell formation (Figure 3), there are lines of evidence for the dynamic nature of the specific subset of cells that are permitted to change, based on timing of prior learning tasks and manipulations of neural excitability [27,28]. Similar changes to the plasticity manifold could result through neuromodulation (reflecting behavioral state of fear, satiety, etc.), metaplasticity, or other pathological conditions.

Degeneracy is the ability of disparate combinations of structural components to perform the same function [59] and provides multiple degrees of freedom to biological systems in achieving functional robustness (Figure 4a). However, the consequent complexity results in parametric variability across animals (or cells or networks), thereby precluding one-to-one relationships between individual components and functional outcomes. The existence of plasticity manifolds represents constraints that restrict unruly changes and therefore provides a valuable handle to probe for order in complex systems manifesting degeneracy.

How do systems (e.g. neurons, networks) expressing degeneracy switch from one valid solution to another toward maintaining functional homeostasis in the face of perturbations? We argue that plasticity manifolds provide a structured substrate for multiple components to change together, thereby seamlessly traversing the valid solution landscape (Figure 4a). Degeneracy implies that for a system in a given state, several plasticity combinations could yield the same function, thereby maintaining functional homeostasis (Figure 4a). Given this, what factors contribute to the system’s ‘decision’ on choosing a specific position on the plasticity manifold versus another (Figure 4a and b)? A critical requirement in systems expressing degeneracy is an error-correcting feedback mechanism that regulates constituent components in achieving a specific function [44,60]. In rhythmogenic circuits where the goal is to maintain specific activity patterns, this feedback signal could be defined as stability of molecular- (e.g. calcium levels), cellular- (e.g. firing rate), or network-scale (e.g. excitation–inhibition balance) physiology. For plasticity manifolds involved in stable learning, however, there is a need to alter the current state of the system toward adapting responses to a novel stimulus (Figure 3b) while still maintaining homeostasis [44]. The feedback signal therefore should convey errors in both stability and learning goals, with learning-related error signals recruiting circuit components implicated in task-dependent sensory or motor feedback [61–63]. These conjunctive feedback signals would then drive the system toward a subset of signaling cascades [27,57,60,64–66], resulting in the choice of a specific plasticity combination (as part of the plasticity manifold) required to achieve stable learning. In addition, degeneracy explains why different systems (performing the same function) react differently to the same perturbation (Figure 4c) and require disparate combinations of plasticity toward achieving stable function (Figure 4a and b) [27,44,57,66–68].

Degeneracy also expresses in the emergence of plasticity manifolds, manifesting as the ability of distinct structural components to yield the same plasticity profile [69], defined as the plasticity rules spanning different values of specific parameters (e.g. calcium-dependent or spike-timing–dependent plasticity profiles). Plasticity degeneracy spans multiple scales, with several possible changes to lower-scale components capable of inducing functional plasticity at a given scale of analysis. For instance, changes in several ion channels could yield similar changes in neuronal firing rate [44]. It is also possible that distinct forms of plasticity, involving different structural components in disparate brain regions, could come together to yield the same learning outcome [27,38,70]. These observations translate to considerable variability in parameters yielding similar plasticity manifolds, implying a lack of one-to-one relationships between individual forms of plasticity and behavioral outcomes. Together, the expression of degeneracy emphasizes the need to account for plasticity manifolds at every scale of analysis, as the rules for emergence of function are distinct across scales [44,71].

## Dynamical nature of plasticity manifolds

Plasticity manifolds are dynamic entities, whereby the rules binding the specific components that undergo conjunctive plasticity could themselves change. A prominent behaviorally relevant route to alter plasticity rules is neuromodulation, a well-established substrate for altering brain states, functional connectivity, and behavior [57,72–74]. Mechanistically, neuromodulation operates by recruiting diverse receptors that activate disparate signaling pathways, with each pathway acting on specific molecular substrates and cellular measurements. Although the impact of neuromodulation in altering synaptic plasticity is well studied [73,74], neuromodulatory regulation of intrinsic plasticity and plasticity manifolds is not fully explored.

The molecular substrates modified by the implementation of the changes that are imposed by a plasticity manifold could alter the plasticity profiles of synapses and neurons. The consequent changes to the rules governing conjunctive changes in several components, including the directions and strengths of such changes, constitute metaplasticity of plasticity manifolds. The mechanistic basis for such metaplasticity could be through changes in synaptic or neuronal properties or through alteration to specific signaling molecules [44,69,75,76]. In the context of stable learning, certain forms of metaplasticity could play a stabilizing role by avoiding run-away excitation. For instance, plasticity in HCN channels [21,22,50,76] and relocation of inhibitory receptors onto synaptic locations [77], both accompanying excitatory synaptic plasticity, have been attributed to stabilizing metaplastic roles.

From the continual learning perspective, one of the several routes to avoid catastrophic forgetting of prior learning [2,78–80] is to ensure that distinct resources (e.g. neurons, ion channel subtypes, or synapses) are allotted for encoding distinct behavioral contexts [18,26,28–31]. Such differential allocation could be achieved if mnemonic plasticity in a subset of resources also introduces concurrent metaplasticity that negatively regulates future recruitment of this subset for other contexts. For instance, TBP recruits a plasticity manifold, inducing suppression of global excitability and concomitant enhancement of local synaptic excitability (Figure 2). Although the localized plasticity specifically enhances the response efficacy of potentiated synapses, the global suppression of excitability ensures that responses to other synaptic inputs are lowered [22] along with a global metaplastic suppression of synaptic potentiation [21,22,50,76]. At the network scale, there is evidence for dynamic resource allocation, established through changes in the subset of cells that are permitted to undergo plasticity toward forming engram cells, based on prior learning tasks and other molecular factors [18,26–28,33].

Plasticity manifolds are recruited and altered by pathological conditions [75,81–94]. An example for the recruitment of plasticity manifolds is repeated stress, where behavioral deficits have been associated with diverse combinations of synaptic, intrinsic, and structural changes in different neurons spanning several brain regions [93–95]. Neurons in animal models of autism spectrum disorders [81 –87] and visual cortical neurons undergoing activity-driven changes induced by visual deprivation [88–91,96] offer examples for altered plasticity manifolds (Figure 4d) involving synaptic (excitatory and inhibitory) and intrinsic plasticity. These structured pathology-driven changes involving plasticity manifolds underscore the need for a holistic approach that measures and incorporates all changes across different brain regions.

## Implications for the existence of plasticity manifolds to computational frameworks and experimental design

The primary implication for the existence of multiscale plasticity manifolds is their ability to sustain stable continual learning in the face of widespread biological heterogeneities, by recruiting disparate components toward efficiently adapting to an ever-changing environment. Learning-theoretical frameworks should incorporate plasticity manifolds, including the synergistic interactions between distinct forms of multiscale plasticity, as a substrate toward stable continual learning (Box 1). Such frameworks for plasticity manifolds could seek inspiration from the well-established neural manifold framework, where the emphasis on conjunctive dynamics of multiple neurons (not just single neurons) continues to provide critical insights on neural encoding [4–7]. Although the neural manifold literature serves as an inspiration, the canvas for plasticity manifolds is much larger (Figures 3 and 4) involving all scales of analyses (from genes to behavior) and all cell types (including all types of neurons and glia).

Experimental designs and technical advances should strongly focus on simultaneously measuring plasticity across cell types in multiple brain regions [70], rather than restricting measurements to changes in a single component (say synaptic strength or neural excitability) in a given brain region. Experimental measurements of multiscale plasticity manifolds are essential because a restricted measurement palette would invariably bias the interpretation on the mechanistic basis of learning-induced adaptation. These measurements of multiscale plasticity and theoretical frameworks on plasticity manifolds could together delineate the functional roles of different components in stable continual learning. Specifically, the changes in components predominantly associated with encoding of the novel environmental context would be attributed a mnemonic role [1]. There would be other components with a homeostatic role toward maintaining stability of multiscale physiology [1]. Furthermore, to sustain the continual nature of the learning process, additional mechanisms could focus on eliminating catastrophic forgetting (e.g. sparse allocation of disparate sets of components to distinct contexts). It is also possible that individual components have different functions under distinct behavioral contexts, whereby plasticity in a specific component might have a homeostatic or a mnemonic or a continual-learning role in distinct contexts.

How do learning-theoretical frameworks and experimental designs account for plasticity manifolds? As the cell type–dependent signaling pathways form the substrate for plasticity manifolds, addressing this requires the entire set of regulatory components in a cell, involving genes, mRNAs, proteins, and metabolites, which has been called the regulome [97]. It is important that techniques are developed to assess the regulome of activity-dependent plasticity in a cell type–dependent manner, evaluating the roles of location and dynamics of different molecular species in the recruitment of specific signaling cascades in yielding plasticity spanning multiple timescales [45,66,98–101]. Theoretical frameworks should then derive rules for plasticity, not just involving synaptic or intrinsic or glial plasticity, but for conjunctive changes in all components of the multiscale manifold involving multiple brain regions to accomplish stable and continual learning.

## Conclusions

Together, learning-theoretical frameworks should build and assess experimentally constrained multiscale models of plasticity manifolds, which are driven by cell type–specific regulomes. Toward achieving stable continual learning, these frameworks should strive to harness (i) the tremendous multiscale computational power of molecular signaling networks, active dendritic structures, and neuron-glia networks spanning different brain regions and (ii) the flexibility and the robustness offered by degeneracy, parametric variability, and neuromodulation (Box 1). The phenomenological and mechanistic insights on the origins of and implications for multiscale plasticity manifolds in biological learning systems could then provide a substrate for incorporating stable continual learning into artificial systems.

## Box 1 How to account for plasticity manifolds in theoretical and experimental studies?

### Molecular scale

- Identify the mechanistic basis for the structure in plasticity manifolds by experimental characterization of cell type–dependent regulome
- Experimentally determine the subcellular loci and temporal evolution of plasticity in molecular components
- Experimentally characterize cell-to-cell variability in signaling molecules and their targets to evaluate heterogeneity in signaling networks and plasticity manifolds through theoretical frameworks
- Decipher rules for molecular-scale plasticity manifolds, accounting for degeneracy and neuromodulation

### Cellular scale

- Identify the functional implications for molecular-scale plasticity manifolds, and define mnemonic, homeostatic, and continual-learning roles for sub-manifolds
- Incorporate active dendritic structures and localized dendritic plasticity into stable continual learning models
- Recognize roles of ion-channel plasticity beyond their roles in regulation of intrinsic excitability (e.g. frequency-dependent filtering, coincidence detection)
- Recognize that plasticity in different components could play distinct roles depending on neuronal subtype, behavioral context, afferent activity, and neuromodulatory tone
- Systematically study metaplasticity of intrinsic plasticity and plasticity manifolds

### Network scale

- Assess molecular- and cellular-scale plasticity manifolds from all neuronal subtypes and glia, accounting for neuromodulation and degeneracy
- Assess stability in network function and probe the implications and mechanisms underlying network stability using conductance-based models
- Implement phenomenological equivalents of plasticity manifolds in neuromorphic hardware that allows for massively parallel computations to assess network-scale plasticity manifolds

### Systems scale

- Characterize and account for feedback loops across different sub-networks, specifically focusing on their roles in driving the system toward achieving stability and learning goals
- Build multiscale models of several interconnected brain regions, each endowed with distinct network architectures. Evaluate the impact of connectivity on activity patterns and plasticity manifolds, while systematically accounting for degeneracy and neuromodulation

## References

[1] Kim SJ, Linden DJ (2007). Ubiquitous plasticity and memory storage. Neuron.

[2] Parisi GI, Kemker R, Part JL, Kanan C, Wermter S (2019). Continual lifelong learning with neural networks: a review. Neural Network.

[3] Turrigiano GG, Nelson SB (2000). Hebb and homeostasis in neuronal plasticity. Curr Opin Neurobiol.

[4] Jazayeri M, Afraz A (2017). Navigating the neural space in search of the neural code. Neuron.

[5] Vyas S, Golub MD, Sussillo D, Shenoy KV (2020). Computation through neural population dynamics. Annu Rev Neurosci.

[6] Gallego JA, Perich MG, Miller LE, Solla SA (2017). Neural manifolds for the control of movement. Neuron.

[7] Chaudhuri R, Gerçek B, Pandey B, Peyrache A, Fiete I (2019). The intrinsic attractor manifold and population dynamics of a canonical cognitive circuit across waking and sleep. Nat Neurosci.

[8] Magee JC, Grienberger C (2020). Synaptic plasticity forms and functions. Annu Rev Neurosci.

[9] Lillicrap TP, Santoro A, Marris L, Akerman CJ, Hinton G (2020). Back-propagation and the brain. Nat Rev Neurosci.

[10] Johnston D, Narayanan R (2008). Active dendrites: colorful wings of the mysterious butterflies. Trends Neurosci.

[11] Stuart GJ, Spruston N (2015). Dendritic integration: 60 years of progress. Nat Neurosci.

[12] Kol A, Goshen I (2020). The memory orchestra: the role of astrocytes and oligodendrocytes in parallel to neurons. Curr Opin Neurobiol.

[13] Ashhad S, Narayanan R (2019). Stores: Channels, glue, and trees: active glial and active dendritic physiology. Mol Neurobiol.

[14] Santello M, Toni N, Volterra A (2019). Astrocyte function from information processing to cognition and cognitive impairment. Nat Neurosci.

[15] Mozzachiodi R, Byrne JH (2010). More than synaptic plasticity: role of nonsynaptic plasticity in learning and memory. Trends Neurosci.

[16] Titley HK, Brunel N, Hansel C (2017). Toward a neurocentric view of learning. Neuron.

[17] Nelson SB, Turrigiano GG (2008). Strength through diversity. Neuron.

[18] Josselyn SA, Tonegawa S (2020). Memory engrams: recalling the past and imagining the future. Science.

[19] Fan Y, Fricker D, Brager DH, Chen X, Lu HC, Chitwood RA, Johnston D (2005). Activity-dependent decrease of excitability in rat hippocampal neurons through increases in I(h). Nat Neurosci.

[20] Lin MT, Lujan R, Watanabe M, Adelman JP, Maylie J (2008). SK2 channel plasticity contributes to LTP at Schaffer collateral-CA1 synapses. Nat Neurosci.

[21] Brager DH, Johnston D (2007). Plasticity of intrinsic excitability during long-term depression is mediated through mGluR-dependent changes in $I_{\rm h}$ in hippocampal CA1 pyramidal neurons. J Neurosci.

[22] Narayanan R, Johnston D (2007). Long-term potentiation in rat hippocampal neurons is accompanied by spatially widespread changes in intrinsic oscillatory dynamics and excitability. Neuron.

[23] Frick A, Magee J, Johnston D (2004). LTP is accompanied by an enhanced local excitability of pyramidal neuron dendrites. Nat Neurosci.

[24] Losonczy A, Makara JK, Magee JC (2008). Compartmentalized dendritic plasticity and input feature storage in neurons. Nature.

[25] Magee JC, Johnston D (1997). A synaptically controlled, associative signal for Hebbian plasticity in hippocampal neurons. Science.

[26] Josselyn SA, Frankland PW (2018). Memory allocation: mechanisms and function. Annu Rev Neurosci.

[27] Sweis BM, Mau W, Rabinowitz S, Cai DJ (2021). Dynamic and heterogeneous neural ensembles contribute to a memory engram. Curr Opin Neurobiol.

[28] Lau JMH, Rashid AJ, Jacob AD, Frankland PW, Schacter DL, Josselyn SA (2020). The role of neuronal excitability, allocation to an engram and memory linking in the behavioral generation of a false memory in mice. Neurobiol Learn Mem.

[29] Zhou Y, Won J, Karlsson MG, Zhou M, Rogerson T, Balaji J, Neve R, Poirazi P, Silva AJ (2009). CREB regulates excitability and the allocation of memory to subsets of neurons in the amygdala. Nat Neurosci.

[30] Lisman J, Cooper K, Sehgal M, Silva AJ (2018). Memory formation depends on both synapse-specific modifications of synaptic strength and cell-specific increases in excitability. Nat Neurosci.

[31] Park A, Jacob AD, Walters BJ, Park S, Rashid AJ, Jung JH, Lau J, Woolley GA, Frankland PW, Josselyn SA (2020). A time-dependent role for the transcription factor CREB in neuronal allocation to an engram underlying a fear memory revealed using a novel in vivo optogenetic tool to modulate CREB function. Neuropsychopharmacology.

[32] Yiu AP, Mercaldo V, Yan C, Richards B, Rashid AJ, Hsiang HL, Pressey J, Mahadevan V, Tran MM, Kushner SA (2014). Neurons are recruited to a memory trace based on relative neuronal excitability immediately before training. Neuron.

[33] Park S, Kramer EE, Mercaldo V, Rashid AJ, Insel N, Frankland PW, Josselyn SA (2016). Neuronal allocation to a hippocampal engram. Neuropsychopharmacology.

[34] Harvey JRM, Plante AE, Meredith AL (2020). Ion channels controlling circadian rhythms in suprachiasmatic nucleus excitability. Physiol Rev.

[35] Patke A, Young MW, Axelrod S (2020). Molecular mechanisms and physiological importance of circadian rhythms. Nat Rev Mol Cell Biol.

[36] Brancaccio M, Edwards MD, Patton AP, Smyllie NJ, Chesham JE, Maywood ES, Hastings MH (2019). Cell-autonomous clock of astrocytes drives circadian behavior in mammals. Science.

[37] Steadman PE, Xia F, Ahmed M, Mocle AJ, Penning ARA, Geraghty AC, Steenland HW, Monje M, Josselyn SA, Frankland PW (2020). Disruption of oligodendrogenesis impairs memory consolidation in adult mice. Neuron.

[38] Ohtsuki G, Shishikura M, Ozaki A (2020). Synergistic excitability plasticity in cerebellar functioning. FEBS J.

[39] Ohtsuki G, Piochon C, Adelman JP, Hansel C (2012). SK2 channel modulation contributes to compartment-specific dendritic plasticity in cerebellar Purkinje cells. Neuron.

[40] Mishra P, Narayanan R (2020). Plasticity manifolds: conjunctive changes in multiple ion channels mediate activity-dependent plasticity in hippocampal granule cells. bioRxiv.

[41] Lee JS, Briguglio JJ, Cohen JD, Romani S, Lee AK (2020). The statistical structure of the hippocampal code for space as a function of time, context, and value. Cell.

[42] Kotaleski JH, Blackwell KT (2010). Modelling the molecular mechanisms of synaptic plasticity using systems biology approaches. Nat Rev Neurosci.

[43] Manninen T, Hituri K, Kotaleski JH, Blackwell KT, Linne ML (2010). Postsynaptic signal transduction models for long-term potentiation and depression. Front Comput Neurosci.

[44] Rathour RK, Narayanan R (2019). Degeneracy in hippocampal physiology and plasticity. Hippocampus.

[45] Bhalla US (2014). Molecular computation in neurons: a modeling perspective. Curr Opin Neurobiol.

[46] Alon U (2019). An introduction to systems biology: design principles of biological circuits.

[47] Ma’ayan A, Jenkins SL, Neves S, Hasseldine A, Grace E, Dubin-Thaler B, Eungdamrong NJ, Weng G, Ram PT, Rice JJ (2005). Formation of regulatory patterns during signal propagation in a Mammalian cellular network. Science.

[48] Neves SR, Iyengar R (2009). Models of spatially restricted biochemical reaction systems. J Biol Chem.

[49] Rosenkranz JA, Frick A, Johnston D (2009). Kinase-dependent modification of dendritic excitability after long-term potentiation. J Physiol.

[50] Honnuraiah S, Narayanan R (2013). A calcium-dependent plasticity rule for HCN channels maintains activity homeostasis and stable synaptic learning. PLoS One.

[51] Shouval HZ, Bear MF, Cooper LN (2002). A unified model of NMDA receptor-dependent bidirectional synaptic plasticity. Proc Natl Acad Sci USA.

[52] Ashhad S, Johnston D, Narayanan R (2015). Activation of InsP3 receptors is sufficient for inducing graded intrinsic plasticity in rat hippocampal pyramidal neurons. J Neurophysiol.

[53] Markram H, Lubke J, Frotscher M, Sakmann B (1997). Regulation of synaptic efficacy by coincidence of postsynaptic APs and EPSPs. Science.

[54] Rahman MM, Kedia S, Fernandes G, Chattarji S (2017). Activation of the same mGluR5 receptors in the amygdala causes divergent effects on specific versus indiscriminate fear. eLife.

[55] Jorntell H, Hansel C (2006). Synaptic memories upside down: bidirectional plasticity at cerebellar parallel fiber-Purkinje cell synapses. Neuron.

[56] Gray JM, Spiegel I (2019). Cell-type-specific programs for activity-regulated gene expression. Curr Opin Neurobiol.

[57] Marder E, Goeritz ML, Otopalik AG (2015). Robust circuit rhythms in small circuits arise from variable circuit components and mechanisms. Curr Opin Neurobiol.

[58] Hamood AW, Marder E (2014). Animal-to-Animal variability in neuromodulation and circuit function. Cold Spring Harbor Symp Quant Biol.

[59] Edelman GM, Gally JA (2001). Degeneracy and complexity in biological systems. Proc Natl Acad Sci U S A.

[60] O’Leary T, Marder E (2016). Temperature-robust neural function from activity-dependent ion channel regulation. Curr Biol.

[61] Ranganathan GN, Apostolides PF, Harnett MT, Xu NL, Druckmann S, Magee JC (2018). Active dendritic integration and mixed neocortical network representations during an adaptive sensing behavior. Nat Neurosci.

[62] Brainard MS, Doupe AJ (2000). Auditory feedback in learning and maintenance of vocal behaviour. Nat Rev Neurosci.

[63] Gadagkar V, Puzerey PA, Chen R, Baird-Daniel E, Farhang AR, Goldberg JH (2016). Dopamine neurons encode performance error in singing birds. Science.

[64] Srikanth S, Narayanan R (2015). Variability in state-dependent plasticity of intrinsic properties during cell-autonomous self-regulation of calcium homeostasis in hippocampal model neurons. eNeuro.

[65] O’Leary T (2018). Homeostasis, failure of homeostasis and degenerate ion channel regulation. Curr Opin Physiol.

[66] Gjorgjieva J, Drion G, Marder E (2016). Computational implications of biophysical diversity and multiple timescales in neurons and synapses for circuit performance. Curr Opin Neurobiol.

[67] Onasch S, Gjorgjieva J (2020). Circuit stability to perturbations reveals hidden variability in the balance of intrinsic and synaptic conductances. J Neurosci.

[68] Marder E, O’Leary T, Shruti S (2014). Neuromodulation of circuits with variable parameters: single neurons and small circuits reveal principles of state-dependent and robust neuromodulation. Annu Rev Neurosci.

[69] Anirudhan A, Narayanan R (2015). Analogous synaptic plasticity profiles emerge from disparate channel combinations. J Neurosci.

[70] Tsutsumi S, Hayashi-Takagi A (2020). Optical interrogation of multiscale neuronal plasticity underlying behavioral learning. Curr Opin Neurobiol.

[71] Anderson PW (1972). More is different. Science.

[72] McCormick DA, Nestvogel DB, He BJ (2020). Neuromodulation of brain state and behavior. Annu Rev Neurosci.

[73] Brzosko Z, Mierau SB, Paulsen O (2019). Neuromodulation of spike-timing-dependent plasticity: past, present, and future. Neuron.

[74] Palacios-Filardo J, Mellor JR (2019). Neuromodulation of hippocampal long-term synaptic plasticity. Curr Opin Neurobiol.

[75] Hulme SR, Jones OD, Abraham WC (2013). Emerging roles of metaplasticity in behaviour and disease. Trends Neurosci.

[76] Narayanan R, Johnston D (2010). The h current is a candidate mechanism for regulating the sliding modification threshold in a BCM-like synaptic learning rule. J Neurophysiol.

[77] Davenport CM, Rajappa R, Katchan L, Taylor CR, Tsai MC, Smith CM, de Jong JW, Arnold DB, Lammel S, Kramer RH (2020). Relocation of an extrasynaptic GABA(A) receptor to inhibitory synapses freezes excitatory synaptic strength and preserves memory. Neuron.

[78] Li Z, Hoiem D (2018). Learning without Forgetting. IEEE Trans Pattern Anal Mach Intell.

[79] Flesch T, Balaguer J, Dekker R, Nili H, Summerfield C (2018). Comparing continual task learning in minds and machines. Proc Natl Acad Sci USA.

[80] Kirkpatrick J, Pascanu R, Rabinowitz N, Veness J, Desjardins G, Rusu AA, Milan K, Quan J, Ramalho T, Grabska-Barwinska A (2017). Overcoming catastrophic forgetting in neural networks. Proc Natl Acad Sci USA.

[81] Huber KM, Gallagher SM, Warren ST, Bear MF (2002). Altered synaptic plasticity in a mouse model of fragile X mental retardation. Proc Natl Acad Sci USA.

[82] Routh BN, Johnston D, Brager DH (2013). Loss of functional A-type potassium channels in the dendrites of CA1 pyramidal neurons from a mouse model of fragile X syndrome. J Neurosci.

[83] Lee HY, Ge WP, Huang W, He Y, Wang GX, Rowson-Baldwin A, Smith SJ, Jan YN, Jan LY (2011). Bidirectional regulation of dendritic voltage-gated potassium channels by the fragile X mental retardation protein. Neuron.

[84] Brager DH, Akhavan AR, Johnston D (2012). Impaired dendritic expression and plasticity of h-channels in the fmr1(-/y) mouse model of fragile X syndrome. Cell Rep.

[85] Peter S, Ten Brinke MM, Stedehouder J, Reinelt CM, Wu B, Zhou H, Zhou K, Boele HJ, Kushner SA, Lee MG (2016). Dysfunctional cerebellar Purkinje cells contribute to autism-like behaviour in Shank2-deficient mice. Nat Commun.

[86] Soda T, Mapelli L, Locatelli F, Botta L, Goldfarb M, Prestori F, D’Angelo E (2019). Hyperexcitability and hyperplasticity disrupt cerebellar signal transfer in the IB2 KO mouse model of autism. J Neurosci.

[87] Tatavarty V, Torrado Pacheco A, Groves Kuhnle C, Lin H, Koundinya P, Miska NJ, Hengen KB, Wagner FF, Van Hooser SD, Turrigiano GG (2020). Autism-associated Shank3 is essential for homeostatic compensation in rodent V1. Neuron.

[88] Maffei A, Nataraj K, Nelson SB, Turrigiano GG (2006). Potentiation of cortical inhibition by visual deprivation. Nature.

[89] Nataraj K, Le Roux N, Nahmani M, Lefort S, Turrigiano G (2010). Visual deprivation suppresses L5 pyramidal neuron excitability by preventing the induction of intrinsic plasticity. Neuron.

[90] Khibnik LA, Cho KK, Bear MF (2010). Relative contribution of feedforward excitatory connections to expression of ocular dominance plasticity in layer 4 of visual cortex. Neuron.

[91] Heynen AJ, Yoon BJ, Liu CH, Chung HJ, Huganir RL, Bear MF (2003). Molecular mechanism for loss of visual cortical responsiveness following brief monocular deprivation. Nat Neurosci.

[92] Beck H, Yaari Y (2008). Plasticity of intrinsic neuronal properties in CNS disorders. Nat Rev Neurosci.

[93] Chattarji S, Tomar A, Suvrathan A, Ghosh S, Rahman MM (2015). Neighborhood matters: divergent patterns of stress-induced plasticity across the brain. Nat Neurosci.

[94] McEwen BS (2007). Physiology and neurobiology of stress and adaptation: central role of the brain. Physiol Rev.

[95] Pignatelli M, Tejeda HA, Barker DJ, Bontempi L, Wu J, Lopez A, Palma Ribeiro S, Lucantonio F, Parise EM, Torres-Berrio A (2020). Cooperative synaptic and intrinsic plasticity in a disynaptic limbic circuit drive stress-induced anhedonia and passive coping in mice. Mol Psychiatr.

[96] Brown APY, Cossell L, Margrie TW (2019). Visual experience regulates the intrinsic excitability of visual cortical neurons to maintain sensory function. Cell Rep.

[97] Townsley KG, Brennand KJ, Huckins LM (2020). Massively parallel techniques for cataloguing the regulome of the human brain. Nat Neurosci.

[98] Uda S, Saito TH, Kudo T, Kokaji T, Tsuchiya T, Kubota H, Komori Y, Ozaki Y, Kuroda S (2013). Robustness and compensation of information transmission of signaling pathways. Science.

[99] Levchenko A, Nemenman I (2014). Cellular noise and information transmission. Curr Opin Biotechnol.

[100] Selimkhanov J, Taylor B, Yao J, Pilko A, Albeck J, Hoffmann A, Tsimring L, Wollman R (2014). Accurate information transmission through dynamic biochemical signaling networks. Science.

[101] Purvis JE, Lahav G (2013). Encoding and decoding cellular information through signaling dynamics. Cell.

